# Comparative Metabolic Characterization of Extraintestinal Pathogenic *Escherichia coli* Blood Isolates from Saudi Arabia

**DOI:** 10.1155/2022/1745835

**Published:** 2022-05-30

**Authors:** Abdulaziz Alangari, Ahmad Abu Jaffal, Abdulaziz M. Alyousef, Abdullah A. Alyousef

**Affiliations:** ^1^Department of Clinical Laboratory Sciences, College of Applied Medical Sciences, King Saud University, P.O. Box 10219, Riyadh 11433, Saudi Arabia; ^2^Department of Clinical Laboratory Sciences, King Saud Bin Abdulaziz University for Health Sciences, Riyadh, Saudi Arabia; ^3^Department of Medical Affairs, Division of Laboratory, Sultan Bin Abdulaziz Humanitarian City, Riyadh, Saudi Arabia

## Abstract

**Background:**

The prevalence of bloodstream infections caused by extraintestinal pathogenic *Escherichia coli* (ExPEC) has increased substantially. E. *coli* ST131 is one of the dominant ExPEC clones among E. *coli* bacteremia population. Metabolism can trigger the pathogenesis of some bacterial isolates, and here we evaluated and compared the metabolic traits of E. *coli* bacteremia isolates including *β*-lactamase (BL)/extended-spectrum *β*-lactamase (ESBL)-positive and ESBL-negative isolates and ST131 and non-ST131 isolates.

**Methods:**

The metabolic profiles of thirty E. *coli* isolates, obtained from blood samples for hospitalized individuals at a tertiary healthcare facility in Riyadh, were determined using HiMedia carbohydrate test strips. The difference in the utilization ability between isolate groups was then statistically assessed.

**Results:**

Our data found that non-BL/ESBL producers were of low metabolic capacity compared with ESBL-positive isolates although the difference remained insignificant. Higher levels of utilization for some carbohydrates, such as fructose and trehalose, were detected among ST131 isolates when compared with non-ST131, and ST131 was also significantly associated with metabolizing rhamnose. The mean bio-score of both isolate groups was insignificant. We showed no link between metabolism and antimicrobial susceptibility profiles among tested blood isolates.

**Conclusion:**

ST131 blood isolates were slightly higher in their carbohydrate utilization activity than non-ST131. More importantly, ST131 isolates were significantly capable of metabolizing rhamnose. Future research should focus on the factors that might drive the success of major ExPEC clones such as ST131.

## 1. Introduction

Bloodstream infections (BSIs), such as bacteremia, are predominantly caused by extraintestinal pathogenic *Escherichia coli* (ExPEC) [1, 2]. The recent escalating number of ExPEC bacteremia cases represents a challenge to global healthcare systems [3, 4]. A previous study found that mortality rates of bacteremia can be as high as 33% in elderly hospitalized patients [5].

The spectrum of ExPEC resistance to several antibiotics, such as trimethoprim-sulfamethoxazole and fluoroquinolones, has increased [6–8]. More recently, many studies have demonstrated the increase in the levels of ExPEC resistance to more powerful agents such as carbapenems and polymyxins [9–12], which can complicate the management of patients. Additionally, some *β*-lactamases (BLs), such as TEM-1 and OXA-1, and extended-spectrum *β*-lactamases (ESBLs), particularly CTX-M family, have highly been detected among ExPEC [13, 14].

E. *coli* sequence type 131 (E. *coli* ST131), discovered in 2008, has been considered the major driver of the current high prevalence of multidrug resistance (MDR) globally [15, 16]. E. *coli* ST131 isolates show insusceptibility to fluoroquinolone (FQ) and carry CTX-M genes, predominantly CTX-M-15 [17–21]. Moreover, it has been shown that ST131 has higher virulence capabilities than other important ExPEC clones [22, 23].

Metabolism is one of many factors that can mediate most of the physiological processes and trigger bacterial pathogenesis. In addition to all types of virulence determinants, such as toxins and adhesions, bacterial pathogens possess specific metabolic traits that allow them to overcome host immune defenses and antimicrobial killing aiding their survival, replication, and colonization [24]. For example, carbohydrate utilization [25] and possessing specific metabolic enzymes [26] can enhance bacterial virulence. A previous study has demonstrated that increased catabolism of D-serine by the E. *coli* ST73 strain, CFT073, can enhance its virulence gene expression during the urinary tract infection process [27]. Additionally, it has been proposed that antimicrobial resistance (AMR) is associated with a high metabolic activity of ExPEC isolates [28]. Another study found that MDR E. *coli* isolates have also been linked with a high ability to utilize particular substrates, such as citrate, as a sole source of carbon [29, 30].

We previously characterized a collection of E. *coli* blood isolates from Saudi Arabia and demonstrated that ST131 accounted for 54.8% of all isolates, 88.2% of which were ESBL-producing [23]. Many previous local and international studies have focused on determining and comparing the metabolic potential of uropathogenic E. *coli* (UPEC) clones [28, 31, 32]. For instance, our research group has recently published a comparative metabolic analysis, on a panel of E. *coli* isolates from urine population, showing no unique metabolic potential of ST131 [31]. However, little is known about metabolic traits of E. *coli* bacteremia population, particularly those for ST131. This comparative study sought to determine the metabolic traits of E. *coli* blood isolates, including BL/ESBL-positive and BL/ESBL-negative and ST131 and non-ST131. It also assessed the relationship between metabolic capacity and antimicrobial resistance of these isolates.

## 2. Materials and Methods

### 2.1. Bacterial Isolates

This study involved using thirty clinical E. *coli* blood isolates. They were part of a larger E. *coli* strain set collected, between January 2018 and March 2018, from bacteremia samples of hospitalized individuals at a main hospital in Riyadh. The isolates were identified as E. *coli* by conventional cultural and biochemical methods. VITEK 2 identification system (VITEK 2-ID-GNB, bioMerieux) was then used for confirmation of preliminary identification and was fully characterized for their antimicrobial sensitivity, BL/ESBL carriage, virulence potential, and ST131 status [22].  Table 1 shows the information on the E. *coli* isolates used in this study.

### 2.2. Metabolic Profiling Assays

KB009 test strips provided on the Hi-Carbohydrate Kit (HiMedia, India) were used to carry out the metabolic profiling of all isolates, according to the manufacturer's instructions. The bacterial metabolic activity was determined by measuring the utilization level of 35 substrates (Table 2). The experiments were carried out in triplicate on two independent occasions showing completely similar results.

### 2.3. Statistical Analysis

IBM SPSS (version 21.0) software was employed to carry out statistical analysis. The metabolism results of different isolate groups were compared using Fisher's exact test (FET). The Mann–Whitney *U* test was used to calculate the mean biochemical scores (mean bio-scores) of isolate groups. The bio-score (BS) was calculated as the sum of all substrates that tested positive for each isolate. The sum of all the BSs of the isolates was then calculated, and finally, this sum was divided by the number of isolates to give the mean bio-score. *p* value of ≤0.05 was used as threshold for statistical significance.

## 3. Results

### 3.1. Metabolic Activity of All E. *coli* Blood Isolates

**Figure 1:**
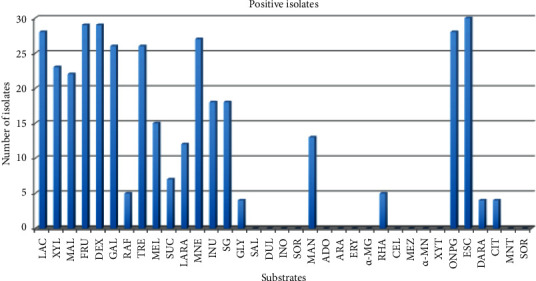
Metabolic activity of all E. *coli* blood isolates.

The metabolic profiling data, obtained from using 35 biochemical substrates, showed that esculin was the only substrate to be utilized by all the tested 30 E*. coli* blood isolates (Figure 1). Nonetheless, all isolates failed to utilize 14 substrates: salicin, dulcitol, inositol, sorbitol, adonitol, arabitol, erythritol, alpha-methyl-D-glucoside, cellobiose, melezitose, alpha-methyl-D-mannoside, xylitol, malonate, and sorbose. Variable utilization levels were found for the remaining 20 substrates such as glycerol, ortho-nitrophenyl-*β*-galactoside, and sucrose (Figure 1). For example, 28 (93.3%) blood isolates were ortho-nitrophenyl-*β*-galactoside-positive, while 2 (6.7%) isolates failed to utilize ortho-nitrophenyl-*β*-galactoside; however, glycerol was utilized by only 4 (13.3%) isolates, whereas 26 (86.7%) isolates did not show activity against glycerol (Figure 1).

### 3.2. The Metabolic Activity of BL/ESBL-Positive and BL/ESBL-Negative E. *coli* Isolates

Our metabolic profiling data showed a complete similarity in the capability of BL/ESBL-positive and BL/ESBL-negative isolates to utilize 21 substrates. Nevertheless, their metabolic activity was variable for the remaining 14 substrates (Table 3). ESBL-producing isolates were higher than non-BL/ESBL producers in metabolizing 9 substrates (fructose, dextrose, trehalose, melibiose, sucrose, mannitol, D-arabinose, citrate, and rhamnose). Additionally, D-arabinose was highly utilized by BL/ESBL-producing members (Table 3). However, BL/ESBL-negative isolates were higher than BL/ESBL producers in utilizing only 5 substrates (xylose, lactose, maltose, raffinose, and mannose).

### 3.3. The Metabolic Activity of E*. coli* ST131 and Non-ST131 Isolates

We found that both isolate groups were similar in metabolizing 16 carbohydrates (Table 4). However, the ability of ST131 isolates in utilizing 13 substrates (maltose, fructose, dextrose, galactose, trehalose, raffinose, sucrose, L-arabinose, mannitol, rhamnose, ortho-nitrophenyl-*β*-galactoside, D-arabinose, and citrate) was higher than non-ST131. Rhamnose was highly utilized by ST131 members compared with non-ST131, and the difference in rhamnose utilization was statistically significant (*p* = 0.04). Nonetheless, non-ST131 isolates were higher than ST131 in utilizing 6 substrates (lactose, xylose, mannose, inulin, sodium gluconate, and glycerol), but this difference remained insignificant (Table 4).

### 3.4. Comparison of the Mean Bio-Scores of E*. coli* Blood Isolate Groups

**Figure 2:**
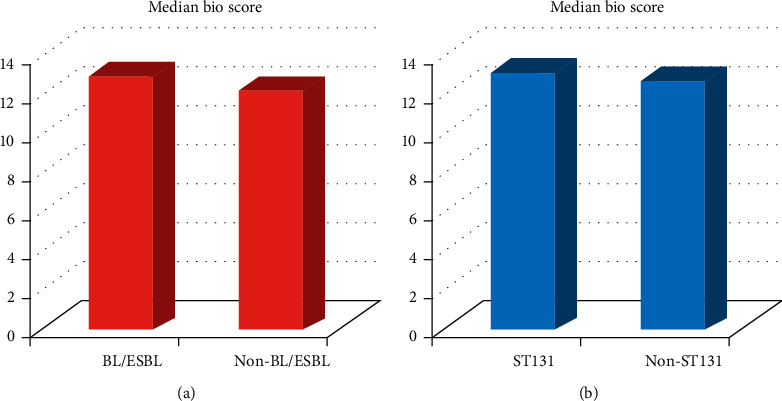
(a) Median bio-scores of BL/ESBL and non-BL/ESBL isolates. The difference between the two isolate groups was insignificant (*p* = 0.77). (b) Median bio-scores of ST131 and non-ST131 isolates. The difference between these isolate groups was insignificant (*p* = 0.48).

The mean bio-scores, defined as the number of substrates, tested positive for an isolate group divided by the total number of tested substrates, of BL/ESBL-positive and ESBL-negative isolates, and ST131 and non-ST131 isolates were determined and compared (Figure 2). Our data demonstrated that the mean bio-score of BL/ESBL producers was 12.73 compared with 12.13 for non-BL/ESBL producers, and the difference was found to be insignificant (*p* = 0.77) (Figure 2). ST131 isolates had a mean bio-score of 12.88, whereas it was 12.64 for non-ST131 (*p* = 0.48) (Figure 2).

### 3.5. Relating the Metabolic Capacity to Antibiotic Susceptibility Profiles of Blood Isolates

Our data found that the non-MDR isolates, B17 and B18, had similar bio-scores compared with the MDR isolates B9 and B10. Moreover, the non-MDR BL/ESBL-negative isolate, B6, had a metabolic bio-score of 6, which was almost comparable to bio-score of the MDR BL/ESBL-producing B29 isolate (Table 5). Additionally, carrying more than one BL/ESBL genotype was not linked to superior metabolic potential in comparison with harboring only one BL/ESBL type. For example, B7, associated with carrying 2 BL/ESBL variants: CTX-M-15 and OXA, had a bio-score of 15, which was lower than that of CTX-M-14-producing isolate B21. Therefore, MDR phenotype and ESBL production were not associated with an elevated metabolic activity among isolates.

## 4. Discussion

Bacteremia due to ExPEC has increasingly been reported in the healthcare and community settings [33, 34]. Metabolism is an important factor used by bacteria to enhance their colonization of hosts [35], and it has been shown that humans could be exploited by many bacterial pathogens as a rich source of nutrients to aid their survival and growth [36]. Given the rapid global dissemination of particular MDR ExPEC clones, such as ST131, previous studies have explored the role of metabolism in driving the success of these clones [28, 31, 32]. However, these reports were limited to UPEC population and the metabolic traits of ExPEC isolates from the bacteremia population have poorly been studied. Here, we determined the metabolic profiles of 30 E*. coli* bacteremia isolates and compared ESBL-positive and ESBL-negative and ST131 and non-ST131 isolates in terms of their metabolic capacity.

Our study found that almost all E*. coli* blood isolates were capable of metabolizing many substrates, such as fructose and lactose. Nonetheless, all isolates entirely failed to utilize many substrates, such as inositol and alpha-methyl-D-glucoside, which is comparable to the typical biochemical activity of E*. coli* for these tests [32]. Interestingly, we reported a reduced utilization activity of our isolates for some substrates in comparison with what is typically known for E*. coli*, particularly those isolated from urine samples. For example, only 43% and 17.6% of these blood isolates were capable of utilizing mannitol and rhamnose, respectively. We previously found very high utilization levels for these two substrates among E*. coli* urine isolates [31]. In this regard, previous comparative phenotypic microarrays analysis of 10 isolates representing the major ExPEC STs, including 190 different substrates, found that bacteremia isolates were associated with lower metabolic activity compared with those isolated from urine [32]. However, given the small sample size used in that study in addition to the very limited number of studies on the metabolic traits of E*. coli* blood isolates, we believe that performing large-scale studies on the metabolic capacity of various ExPEC populations would be essential for providing an accurate comparison between urine and blood isolates in terms of their carbohydrate utilization capabilities.

We also found 4 citrate-positive isolates, and this is surprising given that E*. coli* is usually citrate-negative [37]. This might be ascribed to that some MDR E*. coli* are associated with showing positive citrate reaction [29, 30], which is the case of our blood isolates. In this regard, we have previously demonstrated that 31 of 40 (77.5%) E*. coli* urine isolates were citrate-positive, the majority of which were exhibiting MDR phenotype [31]. Citrate utilization detected among some MDR ExPEC isolates highlights the need to further investigate the factors leading to this unexpected finding.

BL/ESBL-producing blood isolates exhibited higher utilization ability compared with non-BL/ESBL producers; however, no specific metabolic repertoire was detected among BL/ESBL-positive blood isolates. This concurs with the previous finding demonstrating that BL/ESBL production was slightly associated with higher metabolic capabilities among E*. coli* urine isolates [31, 32]. Although some reports have demonstrated the association between AMR and metabolism by which changes in metabolism have a role in modulating bacterial phenotypic resistance to antibiotics [38], it has been shown that AMR is not necessarily linked to metabolism, which might provide an explanation for the absence of specific metabolic profile among our BL/ESBL-producing blood isolates.

Interestingly, our analysis found that ST131 isolates were higher than non-ST131 isolates in metabolizing thirteen substrates and that ST131 was significantly associated with rhamnose utilization. Nevertheless, many studies concluded that ST131 was not the metabolically distinct clone of ExPEC and that no positive association was previously found between ST131 and particular biochemical substrates [31, 32]. However, another study has shown that ST131 urine isolates had higher metabolic profiles compared with other ExPEC STs and that ST131 isolates were positively associated with 5-keto-D-gluconate, beta-glucuronidase, and sucrose [28].

ExPEC isolates can survive in the host bloodstream through many mechanisms. For example, they possess a number of virulence factors, such as group 2 capsular polysaccharide K antigen [39] and increased serum survival (issA) protein [40], allowing them to resist the complement-mediated killing [41]. However, very little is known about the metabolic factors used by ExPEC to trigger their pathogenesis in the bloodstream. In this regard, a recent Chinese analysis, of the mechanisms of porcine ExPEC blood colonization, has identified the upregulation of many genes included in carbon central metabolism, which might play the main role in porcine ExPEC fitness in bloodstream [42]. It is our opinion that the significant association between ST131 and rhamnose shown here merits extensive study in the future to look for the role of this relationship in aiding its survival in the bloodstream.

We found no relationship between antimicrobial resistance and high metabolism potential of the tested blood isolates. This concurs with previous reports showing the same observation among E*. coli* urine population [31, 32]. Nonetheless, Gibreel and coauthors demonstrated that resistant isolates were of higher metabolic potential compared with susceptible isolates [28].

Considering the study limitations, our analysis was performed on bacteremia isolates from one city, Riyadh, which might not be reflective of the metabolic capacity of E*. coli* isolates from other Saudi regions. It also used a relatively low sample size. However, it identified an important finding that shows E*. coli* ST131 blood isolates with an increased metabolic capacity compared with non-ST131.

In conclusion, this is the first study that provides a description on the metabolic traits of E*. coli* blood isolates in Saudi Arabia. We reported that non-BL/ESBL producers were slightly lower in the metabolic capacity compared with ESBL-positive isolates, and this difference was found to be insignificant. Although few insignificant differences in the metabolic capacity were detected between ST131 and non-ST131 isolates, ST131 members were significantly associated with metabolizing rhamnose. Such finding is important and merits further analysis to assess the role of metabolism as a key factor in triggering the pathogenesis of this widespread ExPEC clone.

## Figures and Tables

**Table 1:** Information on the E. *coli* blood isolates used in this study.

Isolate ID	MDR^a^	ESBL	BL/ESBL type(s)	ST131	Reference
B1	MDR	+	TEM-1	+	[23]
B2	Non-MDR	−	NA^b^	−	
B3	MDR	−	NA	−	
B4	MDR	−	NA	+	
B5	MDR	−	NA	−	
B6	Non-MDR	−	NA	−	
B7	MDR	+	CTX-M-15 and OXA-1	+	
B8	MDR	+	CTX-M-15 and TEM-1	+	
B9	MDR	+	OXA-1	+	
B10	MDR	+	CTX-M-15	+	
B11	MDR	−	NA	−	
B12	Non-MDR	−	NA	−	
B13	Non-MDR	−	NA	−	
B14	MDR	−	NA	−	
B15	Non-MDR	−	NA	−	
B16	MDR	+	CTX-M-15 and OXA-1	+	
B17	Non-MDR	−	NA	−	
B18	Non-MDR	−	NA	−	
B19	Non-MDR	−	NA	−	
B20	MDR	+	CTX-M-15 and OXA-1	+	
B21	MDR	+	CTX-M-14	−	
B22	MDR	+	CTX-M-15 and OXA-1	+	
B23	MDR	+	CTX-M-14 and TEM-1	+	
B24	MDR	+	CTX-M-15 and TEM-1	+	
B25	MDR	−	NA	+	
B26	MDR	+	TEM-1	+	
B27	Non-MDR	−	NA	−	
B28	MDR	+	CTX-M-15 and TEM-1	+	
B29	MDR	+	CTX-M-15 and TEM-1	+	
B30	MDR	+	CTX-M-15 and TEM-1	+	

^a^ MDR phenotype refers to displaying resistance to at least 1 antibiotic in ≥3 antibiotic groups. ^b^ NA: not applicable.

**Table 2:** List of substrates used in this study.

Substrates	Abbreviation
Lactose	LAC
Xylose	XYL
Maltose	MAL
Fructose	FRU
Dextrose	DEX
Galactose	GAL
Raffinose	RAF
Trehalose	TRE
Melibiose	MEL
Sucrose	SUC
L-arabinose	LARA
Mannose	MNE
Inulin	INU
Sodium gluconate	SG
Glycerol	GLY
Salicin	SAL
Dulcitol	DUL
Inositol	INO
Sorbitol	SOR
Mannitol	MAN
Adonitol	ADO
Arabitol	ARA
Erythritol	ERY
Alpha-methyl-D-glucoside	*α*-MG
Rhamnose	RHA
Cellobiose	CEL
Melezitose	MEZ
Alpha-methyl-D-mannoside	*α*-MN
Xylitol	XYT
Ortho-nitrophenyl-*β*-galactoside	ONPG
Esculin	ESC
D-arabinose	DARA
Citrate	CIT
Malonate	MNT
Sorbose	SOR

**Table 3:** Metabolic activity of BL/ESBL-producing and non-producing isolates.

Substrates	Positive BL/ESBL-producing isolates (%)	Positive non-BL/ESBL-producing isolates (%)	Total positive isolates (%)	*p* value^a^
LAC	13 (86.7%)	15 (100%)	28 (93.3%)	0.48
XYL	11 (73.3%)	12 (80%)	23 (76.7%)	1.000
MAL	10 (66.7%)	12 (80%)	22 (73.3%)	0.68
FRU	15 (100%)	14 (93.3%)	29 (96.7%)	1.000
DEX	15 (100%)	14 (93.3%)	29 (96.7%)	1.000
GAL	13 (86.7%)	13 (86.7%)	26 (86.7%)	1.000
RAF	2 (13.3%)	3 (20%)	5 (16.7%)	1.000
TRE	14 (93.3%)	12 (80%)	26 (86.7%)	0.59
MEL	8 (53.3%)	7 (46.7%)	15 (50%)	1.000
SUC	4 (26.7%)	3 (20%)	7 (23.3%)	1.000
LARA	6 (40%)	6 (40%)	12 (40%)	1.000
MNE	13 (86.7%)	14 (93.3%)	27 (90%)	1.000
INU	9 (60%)	9 (60%)	18 (60%)	1.000
SG	9 (60%)	9 (60%)	18 (60%)	1.000
GLY	2 (13.3%)	2 (13.3%)	4 (13.3%)	1.000
SAL	0 (0%)	0 (0%)	0 (0%)	1.000
DUL	0 (0%)	0 (0%)	0 (0%)	1.000
INO	0 (0%)	0 (0%)	0 (0%)	1.000
SOR	0 (0%)	0 (0%)	0 (0%)	1.000
MAN	7 (46.7%)	6 (40%)	13 (43.3%)	1.000
ADO	0 (0%)	0 (0%)	0 (0%)	1.000
ARA	0 (0%)	0 (0%)	0 (0%)	1.000
ERY	0 (0%)	0 (0%)	0 (0%)	1.000
*α*-MG	0 (0%)	0 (0%)	0 (0%)	1.000
RHA	4 (26.7%)	1 (6.7%)	5 (16.7%)	0.33
CEL	0 (0%)	0 (0%)	0 (0%)	1.000
MEZ	0 (0%)	0 (0%)	0 (0%)	1.000
*α*-MN	0 (0%)	0 (0%)	0 (0%)	1.000
XYT	0 (0%)	0 (0%)	0 (0%)	1.000
ONPG	14 (93.3%)	14 (93.3%)	28 (93.3%)	1.000
ESC	15 (100%)	15 (100%)	30 (100%)	1.000
DARA	4 (26.7%)	0 (0%)	4 (13.3%)	0.09
CIT	3 (20%)	1 (6.7%)	4 (13.3%)	0.60
MNT	0 (0%)	0 (0%)	0 (0%)	1.000
SOR	0 (0%)	0 (0%)	0 (0%)	1.000

^a^ *p* values for 2-group comparison: ESBL and non-ESBL.

**Table 4:** Metabolic activity of ST131 and non-ST131 isolates.

Substrates	Positive ST131 isolates (%)	Positive non-ST131 isolates (%)	Total positive isolates (%)	*p* value^a^
LAC	14 (87.5%)	14 (100%)	28 (93.3%)	1.000
XYL	12 (75%)	11 (78.6%)	23 (76.7%)	1.000
MAL	12 (75%)	10 (71.4%)	22 (73.3%)	1.000
FRU	16 (100%)	13 (92.9%)	29 (96.7%)	0.47
DEX	16 (100%)	13 (92.9%)	29 (96.7%)	0.47
GAL	14 (87.5%)	12 (85.7%)	26 (86.7%)	1.000
RAF	3 (18.9%)	2 (14.3%)	5 (16.7%)	1.000
TRE	15 (93.8%)	11 (78.6%)	26 (86.7%)	0.32
MEL	8 (50%)	7 (50%)	15 (50%)	1.000
SUC	6 (37.5%)	1 (7.1%)	7 (23.3%)	0.08
LARA	7 (43.8%)	5 (35.7%)	12 (40%)	0.72
MNE	14 (87.5%)	13 (92.9%)	27 (90%)	1.000
INU	9 (56.3%)	9 (64.3%)	18 (60%)	0.72
SG	9 (56.3%)	9 (64.3%)	18 (60%)	0.72
GLY	1 (6.3%)	3 (21.4%)	4 (13.3%)	0.33
SAL	0 (0%)	0 (0%)	0 (0%)	1.000
DUL	0 (0%)	0 (0%)	0 (0%)	1.000
INO	0 (0%)	0 (0%)	0 (0%)	1.000
SOR	0 (0%)	0 (0%)	0 (0%)	1.000
MAN	8 (50%)	5 (35.7%)	13 (43.3%)	0.48
ADO	0 (0%)	0 (0%)	0 (0%)	1.000
ARA	0 (0%)	0 (0%)	0 (0%)	1.000
ERY	0 (0%)	0 (0%)	0 (0%)	1.000
*α*-MG	0 (0%)	0 (0%)	0 (0%)	1.000
RHA	5 (31.3%)	0 (%)	5 (16.7%)	0.04
CEL	0 (0%)	0 (0%)	0 (0%)	1.000
MEZ	0 (0%)	0 (0%)	0 (0%)	1.000
*α*-MN	0 (0%)	0 (0%)	0 (0%)	1.000
XYT	0 (0%)	0 (0%)	0 (0%)	1.000
ONPG	15 (93.4%)	13 (92.9%)	28 (93.3%)	1.000
ESC	16 (100%)	14 (100%)	30 (100%)	1.000
DARA	3 (18.8%)	1 (7.1%)	4 (13.3%)	0.60
CIT	3 (18.8%)	1 (7.1%)	4 (13.3%)	0.60
MNT	0 (0%)	0 (0%)	0 (0%)	1.000
SOR	0 (0%)	0 (0%)	0 (0%)	1.000

^a^ *p* values for 2-group comparison: ST131 and non-ST131, and the bold numbers refer to the presence of significant difference between groups for some traits.

**Table 5:** Relating the antibiotic susceptibility profiles to the biochemical activity of all isolates.

Isolate ID	MDR^a^	ESBL	BL/ESBL type(s)	ST131	Bio-score
B1	MDR	+	TEM-1	+	9
B2	Non-MDR	−	NA^b^	−	11
B3	MDR	−	NA	−	14
B4	MDR	−	NA	+	15
B5	MDR	−	NA	−	9
B6	Non-MDR	−	NA	−	6
B7	MDR	+	CTX-M-15 and OXA-1	+	15
B8	MDR	+	CTX-M-15 and TEM-1	+	14
B9	MDR	+	OXA-1	+	14
B10	MDR	+	CTX-M-15	+	13
B11	MDR	−	NA	−	15
B12	Non-MDR	−	NA	−	14
B13	Non-MDR	−	NA	−	12
B14	MDR	−	NA	−	7
B15	Non-MDR	−	NA	−	13
B16	MDR	+	CTX-M-15 and OXA-1	+	10
B17	Non-MDR	−	NA	−	14
B18	Non-MDR	−	NA	−	13
B19	Non-MDR	−	NA	−	11
B20	MDR	+	CTX-M-15 and OXA-1	+	11
B21	MDR	+	CTX-M-14	-	17
B22	MDR	+	CTX-M-15 and OXA-1	+	12
B23	MDR	+	CTX-M-14 and TEM-1	+	15
B24	MDR	+	CTX-M-15 and TEM-1	+	11
B25	MDR	−	NA	+	17
B26	MDR	+	TEM-1	+	12
B27	Non-MDR	−	NA	−	11
B28	MDR	+	CTX-M-15 and TEM-1	+	19
B29	MDR	+	CTX-M-15 and TEM-1	+	7
B30	MDR	+	CTX-M-15 and TEM-1	+	12

## Data Availability

The data used to support the findings of the study can be obtained from the corresponding author upon reasonable request.

## References

[1] Rogers B. A., Sidjabat H. E., Paterson D. L. (2011). *Escherichia coli* O25b-ST131: a pandemic, multiresistant, community-associated strain. *Journal of Antimicrobial Chemotherapy*.

[2] McNally A., Alhashash F., Collins M. (2013). Genomic analysis of extra intestinal pathogenic *Escherichia coli* urosepsis. *Clinical Microbiology and Infections*.

[3] Poolman J. T., Wacker M. (2016). Extraintestinal pathogenic *Escherichia coli*, a common human pathogen: challenges for vaccine development and progress in the field. *Journal of Infectious Diseases*.

[4] Alhashash F., Weston V., Diggle M., McNally A. (2013). Multidrug-resistant *Escherichia coli* bacteremia. *Emergency Infectious Diseases*.

[5] Tal S., Guller V., Levi S. (2005). Profile and prognosis of febrile elderly patients with bacteremic urinary tract infection. *Journal of Infection*.

[6] Foxman B. (2010). The epidemiology of urinary tract infection. *Nature Review Urology*.

[7] Croxall G., Hale J., Weston V. (2011). Molecular epidemiology of extraintestinal pathogenic *Escherichia coli* isolates from a regional cohort of elderly patients highlights the prevalence of ST131 strains with increased antimicrobial resistance in both community and hospital care settings. *Journal of Antimicrobial Chemotherapy*.

[8] Yezli S., Shibl A. M., Livermore D. M., Memish Z. A. (2014). Prevalence and antimicrobial resistance among gram-negative pathogens in Saudi Arabia. *Journal of Chemotherapy*.

[9] Nordmann P., Naas T., Poirel L. (2011). Global spread of carbapenemase-producing enterobacteriaceae. *Emergency Infectious Diseases*.

[10] Wang R., van Dorp L., Shaw L. P. (2018). The global distribution and spread of the mobilized colistin resistance gene mcr-1. *Nature Communications*.

[11] Alqasim A. (2021). Colistin-resistant gram-negative bacteria in Saudi Arabia: a literature review. *Journal of King Saud University Science*.

[12] Alghoribi M. F., Doumith M., Upton M. (2019). Complete genome sequence of a colistin-resistant uropathogenic *Escherichia coli* sequence type 131 fimH22 strain harboring mcr-1 on an IncHI2 plasmid, isolated in Riyadh, Saudi Arabia. *Microbiol Resour Announc*.

[13] Pitout J. D. D., Nordmann P., Laupland K. B., Poirel L. (2005). Emergence of enterobacteriaceae producing extended-spectrum *β*-lactamases (ESBLs) in the community. *Journal of Antimicrobial Chemotherapy*.

[14] Alqasim A. (2020). Extraintestinal pathogenic *Escherichia coli* in Saudi Arabia: a review of antimicrobial resistance and molecular epidemiology. *Tropical Journal of Pharmaceutical Research*.

[15] Nicolas-Chanoine M. H., Bertrand X., Madec J. Y. (2014). *Escherichia coli* ST131, an intriguing clonal group. *Clinical Microbiology Reviews*.

[16] Price L. B., Johnson J. R., Aziz M. (2013). The epidemic of extended-spectrum-*β*-lactamase-producing *Escherichia coli* ST131 Is driven by a single highly pathogenic subclone, H30-Rx. *mBio*.

[17] Peirano G., Pitout J. D. D. (2010). Molecular epidemiology of *Escherichia coli* producing CTX-M *β*-lactamases: the worldwide emergence of clone ST131 O25:H4. *International Journal of Antimicrobial Agents*.

[18] Nicolas-Chanoine M. H., Blanco J., Leflon-Guibout V. (2008). Intercontinental emergence of *Escherichia coli* clone O25: H4-ST131 producing CTX-M-15. *Journal of Antimicrobical Chemotherapy*.

[19] Alangari A., Jaffal A. A., Almutairi N., Alyousef A. A. (2022). Plasmid replicon diversity of clinical uropathogenic *Escherichia col*i isolates from Riyadh, Saudi Arabia. *Journal of Pure and Applied Microbiology*.

[20] Al-Agamy M. H. (2014). Molecular characteristics of extended-spectrum *β*-lactamase-producing *Escherichia coli* in Riyadh: emergence of CTX-M-15-producing E*. coli* ST131. *Annals Clinical Microbiology Antimicrobial*.

[21] Johnson J. R., Porter S., Thuras P., Castanheira M. (2017). The pandemic H30 subclone of sequence type 131 (ST131) as the leading cause of multidrug-resistant *Escherichia coli* infections in the United States (2011–2012). *Open Forum Infectious Diseases*.

[22] Alqasim A., Abu Jaffal A., Alyousef A. A. (2020). Prevalence and molecular characteristics of sequence type 131 clone among clinical uropathogenic *Escherichia coli* isolates in Riyadh, Saudi Arabia. *Saudi Journal of Biological Sciences*.

[23] Alqasim A., Abu Jaffal A., Almutairi N., Arshad M., Alyousef A. A. (2020). Isolation, phenotypic and genotypic characterization of *Escherichia coli* from the bloodstream samples in Riyadh, Saudi Arabia. *Journal of King Saud University Science*.

[24] Nogales J., Garmendia J. (2022). Bacterial metabolism and pathogenesis intimate intertwining: time for metabolic modelling to come into action. *Microbial Biotechnology*.

[25] Le Bouguénec C., Schouler C. (2011). Sugar metabolism, an additional virulence factor in enterobacteria. *International Journal of Medical Microbiology*.

[26] Pancholi V., Chhatwal G. S. (2003). Housekeeping enzymes as virulence factors for pathogens. *International Journal of Medical Microbiology*.

[27] Anfora A. T., Haugen B. J., Roesch P., Redford P., Welch R. A. (2007). Roles of serine accumulation and catabolism in the colonization of the murine urinary tract by *Escherichia coli* CFT073. *Infection and Immunity*.

[28] Gibreel T. M., Dodgson A. R., Cheesbrough J., Bolton F. J., Fox A. J., Upton M. (2012). High metabolic potential may contribute to the success of ST131 uropathogenic *Escherichia coli*. *Journal of Clinical Microbiology*.

[29] Ishiguro N., Oka C., Sato G. (1978). Isolation of citrate-positive variants of *Escherichia coli* from domestic pigeons, pigs, cattle, and horses. *Applied and Environmental Microbiology*.

[30] Smith H. W., Parsell Z., Green P. (1978). Thermosensitive H1 plasmids determining citrate utilization. *Journal of General Microbiology*.

[31] Alqasim A., Jaffal A. A., Almutairi N., Alyousef A. A. (2021). Comparative phenotypic characterization identifies few differences in the metabolic capacity between *Escherichia coli* ST131 subclones. *Saudi Journal of Biological Sciences*.

[32] Alqasim A., Emes R., Clark G., Newcombe J., La Ragione R., McNally A. (2014). Phenotypic microarrays suggest *Escherichia coli* ST131 Is not a metabolically distinct lineage of extra-intestinal pathogenic E. *coli*. *PloS One*.

[33] Riley L. (2014). Pandemic lineages of extraintestinal pathogenic *Escherichia coli*. *Clinical Microbiology and Infections*.

[34] Alhashash F., Wang X., Paszkiewicz K., Diggle M., Zong Z., McNally A. (2015). Increase in bacteraemia cases in the East Midlands region of the UK due to MDR *Escherichia coli*ST73: high levels of genomic and plasmid diversity in causative isolates. *Journal of Antimicrobial Chemotherapy*.

[35] Rohmer L., Hocquet D., Miller S. I. (2011). Are pathogenic bacteria just looking for food? metabolism and microbial pathogenesis. *Trends in Microbiology*.

[36] Costantini C., Bellet M. M., Renga G. (2020). Tryptophan co-metabolism at the host-pathogen interface. *Frontiers in Immunology*.

[37] Reynolds C. H., Silver S. (1983). Citrate utilization by *Escherichia coli*: plasmid-and chromosome-encoded systems. *Journal of Bacteriology*.

[38] Martínez J. L., Rojo F. (2011). Metabolic regulation of antibiotic resistance. *FEMS Microbiology Reviews*.

[39] Whitfield C. (2006). Biosynthesis and assembly of capsular polysaccharides in *Escherichia coli*. *Annual Review of Biochemistry*.

[40] Biran D., Sura T., Otto A., Yair Y., Becher D., Ron E. Z. (2021). Surviving serum: the *Escherichia coli* iss gene of extraintestinal pathogenic E. *coli* is required for the synthesis of group 4 capsule. *Infection and Immunity*.

[41] Miajlovic H., Cooke N. M., Moran G. P., Rogers T. R. F., Smith S. G. (2014). Response of extraintestinal pathogenic *Escherichia coli* to human serum reveals a protective role for Rcs-regulated exopolysaccharide colanic acid. *Infection and Immunity*.

[42] Ma J., Pan X., Zhong X., Bai Q., Liu G., Yao H. (2020). Preferential use of carbon central metabolism and anaerobic respiratory chains in porcine extraintestinal pathogenic *Escherichia coli* during bloodstream infection. *Veterinary Microbiology*.

